# Diagnostic Potential of Novel Salivary Host Biomarkers as Candidates for the Immunological Diagnosis of Tuberculosis Disease and Monitoring of Tuberculosis Treatment Response

**DOI:** 10.1371/journal.pone.0160546

**Published:** 2016-08-03

**Authors:** Ruschca Jacobs, Elizna Maasdorp, Stephanus Malherbe, Andre G. Loxton, Kim Stanley, Gian van der Spuy, Gerhard Walzl, Novel N. Chegou

**Affiliations:** DST/NRF Centre of Excellence for Biomedical Tuberculosis Research and SAMRC Centre for Tuberculosis Research, Division of Molecular Biology and Human Genetics, Department of Biomedical Sciences, Faculty of Medicine and Health Sciences, Stellenbosch University, Cape Town, South Africa; McGill University, CANADA

## Abstract

**Background:**

There is an urgent need for new tools for the early diagnosis of TB disease and monitoring of the response to treatment, especially in resource-constrained settings. We investigated the usefulness of host markers detected in saliva as candidate biomarkers for the immunological diagnosis of TB disease and monitoring of treatment response.

**Methods:**

We prospectively collected saliva samples from 51 individuals that presented with signs and symptoms suggestive of TB disease at a health centre in Cape Town, South Africa, prior to the establishment of a clinical diagnosis. Patients were later classified as having TB disease or other respiratory disease (ORD), using a combination of clinical, radiological and laboratory findings. We evaluated the concentrations of 69 host markers in saliva samples using a multiplex cytokine platform, and assessed the diagnostic potentials of these markers by receiver operator characteristics (ROC) curve analysis, and general discriminant analysis.

**Results:**

Out of the 51 study participants, 18 (35.4%) were diagnosed with TB disease and 12 (23.5%) were HIV infected. Only two of the 69 host markers that were evaluated (IL-16 and IL-23) diagnosed TB disease individually with area under the ROC curve ≥0.70. A five-marker biosignature comprising of IL-1β, IL-23, ECM-1, HCC1 and fibrinogen diagnosed TB disease with a sensitivity of 88.9% (95% CI,76.7–99.9%) and specificity of 89.7% (95% CI, 60.4–96.6%) after leave-one-out cross validation, regardless of HIV infection status. Eight-marker biosignatures performed with a sensitivity of 100% (95% CI, 83.2–100%) and specificity of 95% (95% CI, 68.1–99.9%) in the absence of HIV infection. Furthermore, the concentrations of 11 of the markers changed during treatment, indicating that they may be useful in monitoring of TB treatment response.

**Conclusion:**

We have identified novel salivary biosignatures which may be useful in the diagnosis of TB disease and monitoring of the response to TB treatment. Our findings require further validation in larger studies before these biosignatures could be considered for point-of-care screening test development.

## Introduction

Tuberculosis (TB) remains a global health problem. According to the World Health Organisation (WHO), 1.5 million people died from the disease in 2014 [1]. The global TB epidemic continues to in part be driven by undiagnosed TB cases or delays in the diagnosis of the disease, which results in delays in treatment initiation and increases chances of transmission. Therefore the need for rapid and accurate tools for both the diagnosis and monitoring of TB treatment response remains a priority for the global control of the disease. Current diagnostic tools have several drawbacks, including the low sensitivity of the Ziehl Neelsen smear microscopy test and the unavailability and long turn-around time of the current gold standard (culture). Furthermore the long turn-around time of culture limits its use as a means to monitor the response to TB treatment [2,3]. The diagnosis of TB disease has significantly improved with the roll-out of the automated gene amplification test GeneXpert (Cepheid Inc., Sunnyvale, USA), as the test greatly reduces the time to detection and is coupled with the identification of resistance to rifampicin. However this test is costly and requires infrastructure that is not readily available in resource constrained settings, and is therefore not ideal in these areas [4]. Immunodiagnostic approaches might be beneficial especially if based on more easily available sample types such as saliva, whole blood, plasma or serum, for both the diagnosis of TB disease and monitoring of treatment response. The relatively easier adaptability of host biomarker-based tests into rapid point-of-care tests, makes them very promising for resource-constrained settings [5]. Additionally, such tests may be useful particularly in circumstances where sputum collection is difficult, for example, in paediatric TB, and in paucibacillary forms of the disease such as extra-pulmonary TB and co-infection with HIV.

Interferon gamma (IFN-γ) release assays (IGRAs) and the tuberculin skin test remain the most widely used commercially available TB immunodiagnostic tests. The use of IGRAs is however limited in high TB endemic areas as these assays are not useful in the diagnosis of active TB disease, which is a major problem in these areas with high prevalence of latent infection [6]. IGRAs have also generated inconsistent results as tools for monitoring of the response to TB treatment [7,8]. Host markers other than IFN-γ detected after overnight stimulation with the antigens employed in IGRAs (ESAT-6/CFP-10/TB7.7) and markers produced after stimulation with novel *M*.*tb* infection phase dependent antigens have shown promise [9,10]. However overnight culture-based assays are unable to serve as rapid, point-of-care tests. Host biomarkers detected in *ex vivo* samples such as serum, plasma, saliva and other effusions have shown potential in the diagnosis of TB disease [11–14]. Although saliva has been shown to be an important diagnostic fluid in numerous diseases, including systemic, oral infections, and HIV [15], not much has been done on this potentially valuable sample type in the TB field. Saliva is an easily obtainable sample and can be collected non-invasively with limited training and basic equipment. It is abundantly available in all individuals and an average adult has been reported to always have about 1ml of saliva in the oral cavity [16]. Recent studies have shown an up to 6-fold higher expression of some host biomarkers in saliva when compared to serum samples from TB patients [12], and that some of these host markers may be useful as tools for the diagnosis of TB disease and monitoring of the response to treatment [11,12]. Given the potential that diagnostic tools based on host biomarkers detected in saliva might have in the control of TB disease, it is important to continue investigating new host biomarkers in this potentially valuable sample type, in an attempt to identify better diagnostic candidates, and to refine the markers that have shown potential in previous studies, pending validation in larger prospective studies. In a recent large multi-centered study conducted on serum samples [17], combinations between new and established host biomarkers showed potential in the diagnosis of TB disease. As most of the new candidate biomarkers have not been previously investigated in saliva, we aimed to investigate the diagnostic utility of salivary levels of these host markers for TB disease and to ascertain whether any of these markers were potentially useful in monitoring of the response to TB treatment. We also investigated the utility of biomarkers that showed potential as salivary diagnostic candidates in recently published studies.

## Materials and Methods

### Study participants

Participants included in the current study were recruited from the Fisantekraal Community Clinic, in the outskirts of Cape Town, South Africa, as part of a previously reported large study; the African European tuberculosis Consortium (AE-TBC; www.ae-tbc.eu) [11,17]. As described previously [11,17], all study participants presented with signs and symptoms suggestive of TB disease, including persistent cough lasting ≥2 weeks and at least one of either fever, malaise, recent weight loss, night sweats, knowledge of close contact with a TB patient, haemoptysis, chest pain or loss of appetite, and were recruited prior to clinical or laboratory assessment for TB disease. Participants were eligible for the study if they were 18 years or older and willing to give written informed consent for participation in the study, including consent for HIV testing. Patients were excluded if they were pregnant, had not been residing in the study community for more than 3 months, were severely anaemic (haemoglobin <10 g/l), were on anti-TB treatment, had received anti-TB treatment in the previous 90 days or if they were on quinolone or aminoglycoside antibiotics during the past 60 days. Recruitment of study participants was done between November 2010 and November 2012. The study was approved by the Health Research Ethics Committee of the Faculty of Medicine and Health Sciences of the University of Stellenbosch and written informed consent was obtained from all participants.

### Sample collection and diagnostic tests

Study participants fasted for at least one hour before saliva collection. Briefly, participants were asked to chew a sterile cotton swab (salivette) that was provided by the saliva collection kit manufacturer (Sarstedt, Numbrecht, Germany), for about 45 seconds. The swab was then removed from the participant’s mouth with sterile forceps, inserted into a sterile tube provided by the manufacturer, and then transported to the laboratory at 4–8°C. Upon arrival in the laboratory, the saliva samples were centrifuged at 1000g for 2 minutes and the supernatant harvested and stored at -80°C until tested. After microbiological confirmation of TB disease in study participants, sample collection was repeated for the culture confirmed TB patients at month 2 and month 6 after the initiation of TB treatment. As previously described [11], sputum samples collected from all study participants were cultured using the MGIT method (BD Biosciences, Franklin Lakes, NJ, USA), after which positive MGIT cultures were examined for acid fast bacilli using the Ziehl-Neelsen technique (to check for contamination), followed by Capilia TB testing (TAUNS, Numazu, Japan), to confirm the isolation of organisms of the *M*.*tb* complex, before being designated as positive cultures.

### Classification of study participants and reference standard

As previously described [11], participants were classified as definite TB patients, probable TB patients, participants with other respiratory diseases (ORD) or questionable disease, using a combination of clinical, radiological, and laboratory findings. For the present discovery study however, only definite (culture positive) TB patients were included. Using an online tool (www.random.org), we then randomly selected individuals with ORD (n = 33) for investigation alongside the definite TB patients with available saliva samples (n = 18), due to the considerably higher numbers of individuals with ORD in our biobank. As described previously [17], individuals with ORD had a range of other diagnoses, including upper and lower respiratory tract infections (viral and bacterial infections, although attempts to identify organisms by bacterial or viral cultures were not made), and acute exacerbations of chronic obstructive pulmonary disease or asthma. Such investigations are not routinely done at primary health care settings, for example, where study participants were recruited.

### Luminex Multiplex Immunoassay

The concentrations of 69 host markers, including markers that have not been widely investigated in the TB field (NCAM, transhyretin, MIP-4, antithrombin-III, GDF-15, ADAMTS13, (in kits purchased from Merck Millipore, Billerica, MA, USA), and other host markers namely: alpha2 macroglobulin (A2M), haptoglobin, C-reactive protein (CRP), serum amyloid P (SAP), procalcitonin (PCT), ferritin, tissue plasminogen activator (TPA), fibrinogen, serum amyloid A (SAA) (in kits purchased from Bio- Rad Laboratories, Hercules, CA, USA), vitronectin, extracellular matrix protein 1 (ECM1), vitamin D binding protein, sFas, granzyme A, sFasL, sCD137, granzyme_B, perforin, myoglobulin, P-selectin, lipocalin-2, thrombopoietin (TPO), stem cell factor (SCF), B-cell attracting chemokine 1 (BCA-1), epithelial neutrophil activating protein (ENA)-78, thymic stromal lymhpopoietin (TSLP), I-309(CCL-1), stromal cell derived factor 1 alpha (SDF-1α), IFN-γ, IFN-α 2, interferon inducible protein (IP)-10, macrophage inflammatory protein (MIP)-1β, tumor necrosis factor (TNF)-α, TNF-β, vascular endothelial growth factor (VEGF), soluble CD40 ligand (sCD40L), apolipoprotein (Apo) A-1, Apo CIII, complement component 3, complement factor H (CFH), total plasminogen activator inhibitor 1 (PAI-1), brain-derived neurotrophic factor (BDNF), cathepsin D, myeloperoxidase (MPO), matrix metalloproteinase (MMP)-2, MMP-9, hemofiltrate CC chemokine 1 (HCC-1), α-1-antitrypsin, pigment epithelium derived factor (PEDF), complement C4, interleukin (IL)-17F, IL-17A, IL-22, IL-33, IL-21, IL-23, IL-25, IL-31, IL-28A, IL-16, IL-1β, IL-12(p40) and IL-13 (Merck Millipore, Billerica, MA, USA), were investigated in saliva samples from all the study participants. Experiments were performed in a blinded manner, according to the instructions of the kit manufacturers. All assays were read on the Bio-Plex platform (Bio-Rad), with the Bio-Plex Software version 6.1 used for bead acquisition and analysis.

### Statistical Analysis

Differences in the concentrations of host markers detected in saliva samples from TB patients and individuals with ORD were evaluated using the Mann-Whitney U test, for non-normally distributed data, whereas the student’s t-test was used if data were normally distributed. The diagnostic abilities of individual host markers were investigated by receiver operator characteristics (ROC) curve analysis. The cut-off values for each analyte were determined by selecting the maximum values of Youden’s index [18]. General discriminant analysis (GDA) was used to determine the predictive abilities of combinations of markers for the diagnosis of TB disease, with leave one-out cross validation [19]. Differences in the expression profiles of host markers during the course of TB treatment were analysed by mixed model repeated measures analysis of variance (ANOVA), with Fisher’s Least Significant Difference (LSD) post hoc testing. P-values ≤0.05 were considered significant. The data were analysed using Statistica (Statsoft, Ohio, USA) and Graphpad Prism version 5 (Graphpad Software Inc., CA, USA).

## Results

A total of 51 study participants, 18 of whom were culture positive TB patients were investigated in this study. Twelve (23.5%) of the study participants were HIV infected. The clinical and demographic characteristics of study participants are shown in Table 1.

**Table 1:** Clinical and demographic characteristics of study participants.

Number of participants	All (n = 51)	TB (n = 18)	ORD (n = 33)
Male, n (%)	20 (39)	4 (22)	16 (48)
Mean age, (Years)±SD	36.4 ± 9.8	39.1 ± 9.7	35 ± 9.7
HIV infected, n (%)	12 (24)	4(22)	8 (24)
Quantiferon results			
Positive, n (%)	30 (60)	12 (71)	18 (55)
Negative, n (%)	19 (38)	4 (24)	15 (45)
Indeterminate, n (%)	1 (2)	1 (6)	0 (0)

All the 18 TB patients included in the study were culture confirmed.

TB = pulmonary tuberculosis, SD = standard deviation

### Utility of individual salivary host markers in the diagnosis of TB disease

**Fig 1:**
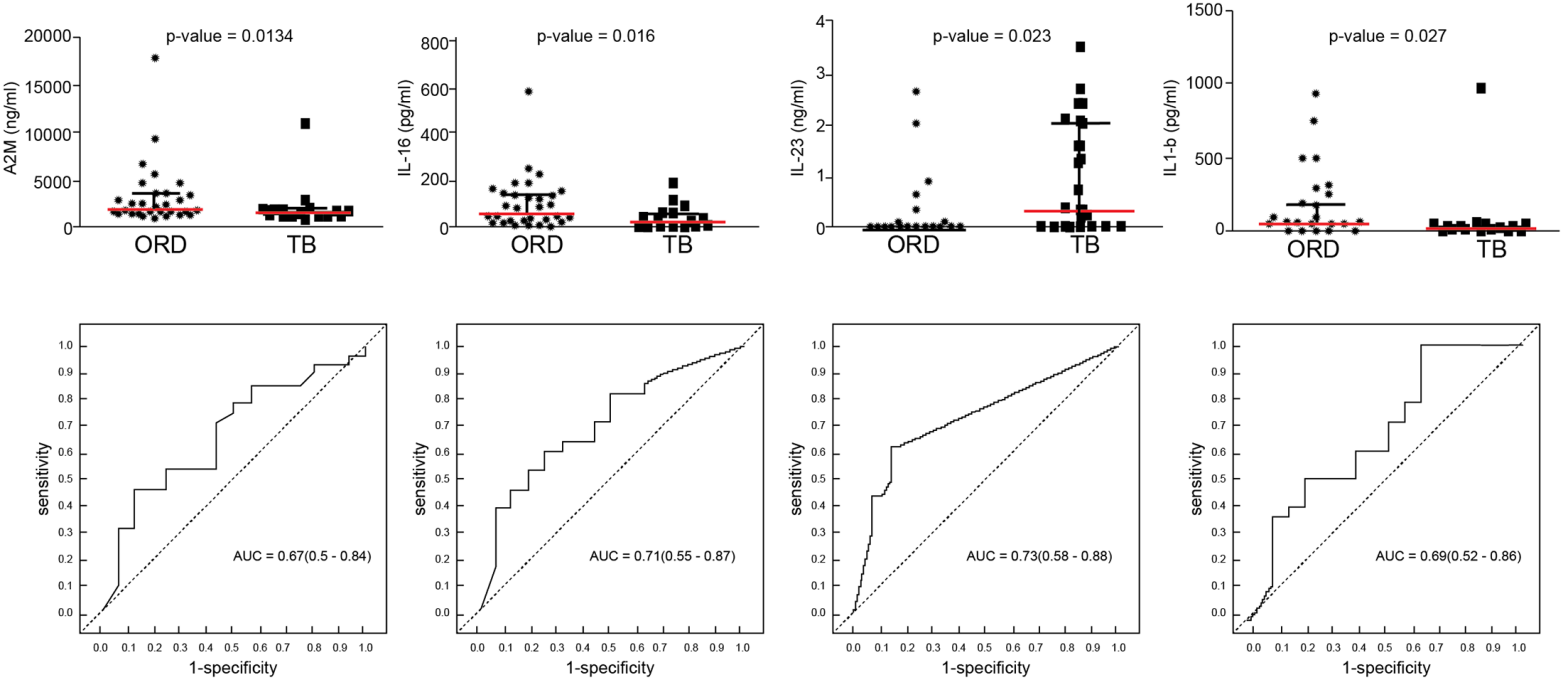
Scatter plots showing the concentrations of host markers detected in saliva samples from TB patients (n = 18) and individuals with ORD (n = 33) and receiver operator characteristics curves showing the accuracies of these markers in the diagnosis of TB disease. Representative plots are shown for A2M, IL-16, IL-23, and IL-17A. Error bars in the scatter dot plots represent the median with interquartile range.

Of the 69 markers investigated in the current study, 23 (vitronectin, vitamin D binding protein, sFas, sFasL, sCD137, perforin, ADAMTS13, P-selectin, SCF, IL-28, Apo A-1, Apo CIII, CFH, NCAM, BDNF, MMP-2, MIP-4, complement C4, IL-17F, IL-25 and IL-23, IL-22, IL-33) were barely or not detectable in saliva samples and these markers were excluded from further analysis. When the baseline concentrations of the remaining 46 markers in the TB patients (n = 18) were compared to the levels obtained in the 33 individuals with ORD with the Mann Whitney U or student’s t test, only eight host markers were significantly different between the two groups. The median levels of IL-17A, IL-23 and the mean of concentrations of ECM-1 were significantly higher in the TB patients, whereas the median levels of A2M, SAP, IL-16, IL-1β, and the mean levels of granzyme B were significantly higher in the ORD group, whereas trends towards higher levels of myoglobulin and GDF-15 were observed in TB patients (Table 2). When the diagnostic accuracies of individual host markers were investigated by ROC curve analysis, only two markers (IL-16 and IL-23) showed promise in the present study with area under the ROC curve (AUC) ≥0.70, whereas A2M, SAP, IL-17A and IL-1β performed with AUCs ≥ 0.67 (Table 2). Two additional markers (GDF-15 and IL-25) became significantly different between the TB patients and individuals with ORD, and/or showed diagnostic potential (AUCs of 0.72 and 0.53 respectively) only after data for HIV infected individuals were excluded. Representative plots showing the levels of some of the individual host markers with diagnostic potential, regardless of HIV infections status are shown in Fig 1.

**Table 2:** Median levels and interquartile ranges (in parenthesis) of host markers detected in baseline saliva samples from the TB patients (n = 18) and individuals with ORD (n = 33) and their diagnostic accuracies for TB disease.

Marker	ORD	TB Disease	P value	AUC	Cut off value	Sensitivity %(95% CI)	Specificity %(95% CI)
A2M	1966(1546–3503)	1390(1168–1926)	0.013	0.67(0.5–0.84)	< 1351	50(26–74)	88 (72–97)
ECM-1^a^	14.6±14.4	22.3±12.4	0.046	0.66(0.49–0.84)	> 15.43	83(59–96)	48 ((31–66)
GDF-15	0.02(0.02–0.1)	0.05(0.02–0.08)	0.088	0.64(0.49–0.80)	> 0.0550	50(26–74)	76 (58–89)
Granzyme B^a^	0.02±0.05	0.001±0.003	0.036	0.55	< 0.0150	100 (81–100)	27 (13–46)
IL-1β	36.4(15.9–183.4)	16.9(4.3–39.4)	0.027	0.69(0.52–0.86)	< 7.285	39(17–64)	97 (84–100)
IL-16	56.1(22.0–144.3)	20.01(0–59.39)	0.016	0.71(0.55–0.87)	< 14.23	50(26–74)	85 (68–95)
IL-17A	6.1(2.2–10)	13.8(3.9–29.4)	0.028	0.69(0.53–0.85)	> 8.035	72(47–90)	67 (48–82)
IL-23	0(0–0)	0.3(0–2.0)	0.0023	0.73(0.59–0.88)	> 0.1550	61(36–83)	85 (68–95)
Myoglobulin	0.4(0.2–0.7)	0.6(0.3–0.8)	0.097	0.64(0.49–0.80)	>0.2500	100(81–100)	27 (13–46)
SAP	61.0(5.5–61.0)	5.5(5.5–5.5)	0.031	0.67(0.51–0.82)	<33.26	83(59–96)	52 (34–69)

Only markers that showed significant differences or trends between groups with the Mann-Whitney U test are shown. The concentrations of A2M, SAP, ECM1, IL-23, myoglobulin and GDF-15 are in ng/ml. The concentrations of all the other markers are in pg/ml.

^a^Mean ± standard deviation. Data for these host markers were normally distributed

### Utility of multi-saliva marker combinations in the diagnosis of TB disease

When the data obtained from all study participants were fitted into General Discriminant Analysis (GDA) models, regardless of HIV status, combinations between up to five different host markers showed potential in the diagnosis of TB disease. A three-marker biosignature comprising of granzyme B, fibrinogen and A2M diagnosed TB disease with a sensitivity of 94.4% (95% CI, 74.0–99.9%) and specificity of 62.1% (95% CI, 40.6–78.5%) in the resubstitution classification matrix and sensitivity of 94.4% (95% CI,72.7–99.9%) and specificity of 58.6% (95% CI, 38.9–76.5%) after leave-one-out cross validation. However, the most optimal diagnostic biosignature irrespective of HIV status was a combination between five markers (IL-1β, IL-23, ECM-1, HCC1 and fibrinogen) which diagnosed TB disease with a sensitivity 88.9% (95% CI,76.7–99.9%) and specificity of 89.7% (95% CI, 60.4–96.6%) in the resubstitution classification matrix, and with the same accuracy (sensitivity of 88.9% and specificity of 89.7%) after leave-one-out cross validation. The positive and negative predictive values of the five-marker biosignature were 89.7% (95% CI, 76.7–97.8%) and 88.9% (95% CI, 65.3–98.6%) respectively.

**Fig 2:**
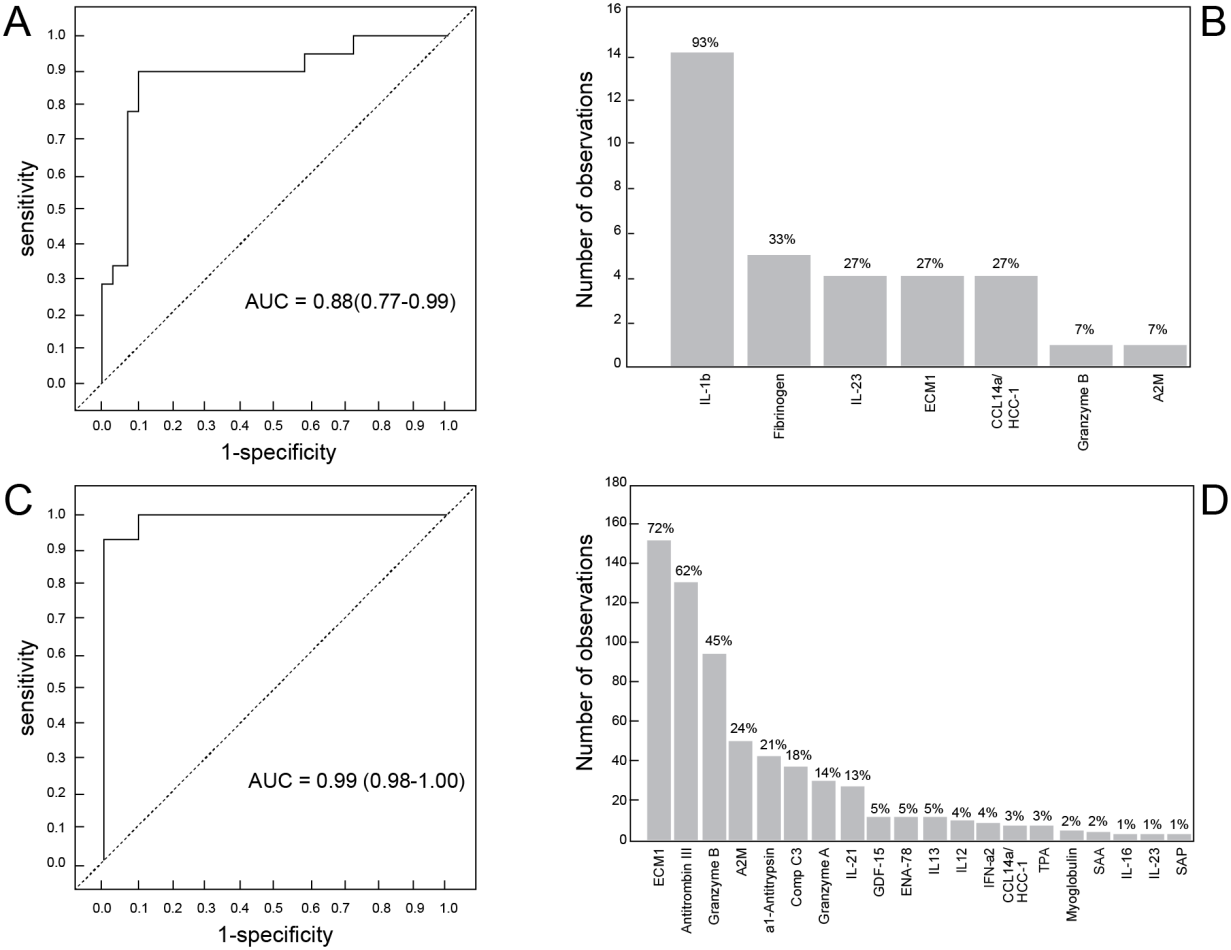
Accuracy of salivary multi-marker models in the diagnosis of TB disease. Receiver operator characteristics (ROC) curve showing the accuracy of the optimal five-marker biosignature (IL-1β, IL-23, ECM1, HCC1, fibrinogen) in the diagnosis of TB disease in all study participants, regardless of HIV infection status (A), frequency of analytes in top general discriminant analysis (GDA) models that most accurately classified individuals as TB patients or ORD irrespective of HIV status (B), ROC curve showing the accuracy of the most accurate eight-marker biosignature (granzyme A+PAI-1+GDF-15+SAA+IL-21+ENA-78+IL-12(p40) and IL-13) in the diagnosis of TB disease after exclusion of HIV positive individuals (C), and frequency of analytes in the top GDA models that most accurately classified study participants as TB or ORD in the absence of HIV infection (D). The bar graphs (B and D) indicate the frequency of analytes in the most accurate GDA models.

When the GDA procedure was repeated after excluding the HIV infected individuals, two eight-marker biosignatures diagnosed TB disease with high accuracies. A biosignature comprising of granzyme A, GDF-15, SAA, IL-21, ENA-78, IL-12(p40), IL-13 and PAI-1,diagnosed TB disease with sensitivity of 93% (95% CI, 77.2–99.9%) and specificity of 100% (95% CI, 75.3–100%) in the resubstitution classification matrix, and after leave-one-out cross validation, whereas a biosignature comprising of ECM1, myoglobulin, HCC1, IL-21, ENA-78, TPA, IL-12(p40) and IL-13 diagnosed TB disease with sensitivity of 100% (95% CI, 83.2–100%) and specificity of 95% (95% CI, 68.1–99.9%) in the resubstitution classification matrix, and after leave-one-out cross validation. IL-1β was the most frequent analyte in biosignatures, appearing in 93% of the biosignatures generated for diagnosing TB disease regardless of HIV infection status (Fig 2).

### Changes in the concentrations of host biomarkers during the course of TB treatment

**Fig 3:**
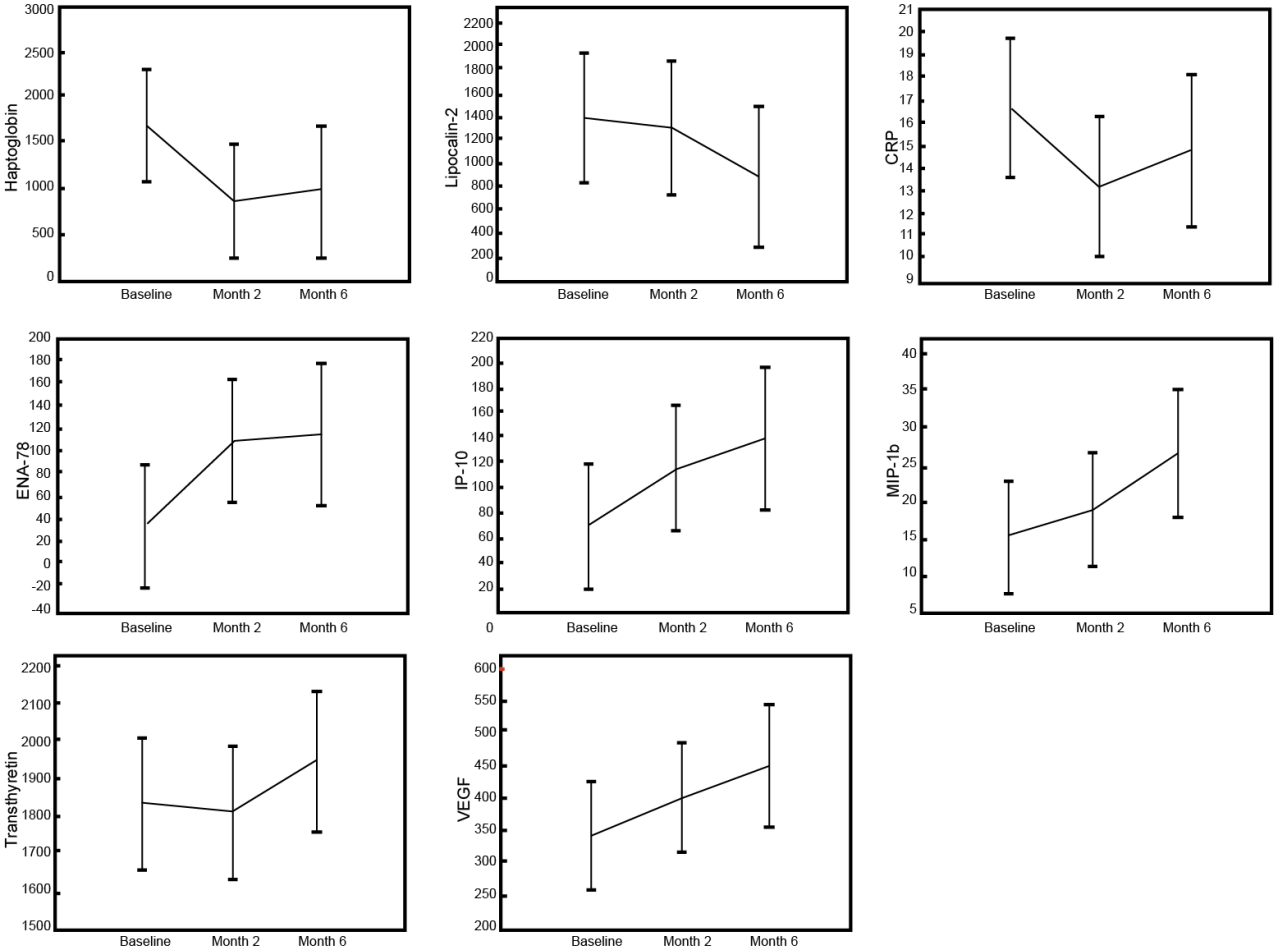
Changes in the concentrations of host markers in saliva samples from TB patients undergoing TB treatment. Saliva samples were collected from all study participants at baseline (before the start of treatment) and at months 2 and 6 after initiation of TB treatment. Error bars indicate the Least Squared means with 95% Confidence Intervals.

To investigate whether any of the 46 detectable host markers could potentially be used as markers to monitor TB treatment response, we evaluated the concentrations of the markers in saliva samples that were collected from TB patients at baseline, and at months 2 and 6, following the start of TB treatment. Of the 18 TB patients included in the study, all 18 (100%) and 13 (72%) returned to the clinic and provided samples at months 2 and 6 respectively, and these samples were used in this part of the study. The salivary concentrations of 8 host markers changed significantly during the course of treatment. There was a significant decrease in the levels of haptoglobin and CRP from baseline to month 2, but a significant increase in ENA-78 levels over the same period (Fig 3, Table 3). The levels of ENA-78 continued to increase towards month 6. The levels of IP-10, MIP-1β and VEGF increased significantly when baseline levels were compared to end of treatment (month 6) levels, whereas the levels of lipocalin-2 significantly decreased and a trend towards decreasing levels of ECM1 from baseline to month 6 was observed (Fig 3, Table 3). There were no significant differences in transthyretin levels between diagnosis and month 2, but the increase in the concentrations of the protein became significant by the end of treatment (Fig 3, Table 3).

**Table 3:** Mean values of host makers detected in saliva samples of TB patients at baseline, month 2 and month 6 (after the start of TB treatment).

	Time point			P value		
Marker	Baseline	Month 2	Month 6	Baseline Vs. M2	M2 Vs. M6	Baseline Vs. M6
**CRP**	16 (12.7–19.5)	13.2 (9.8–16.6)	14.2 (9.9–18.5)	**0.015**	0.27	0.21
**ECM-1**	22 (15.9–28.2)	15.5 (8.4–22.6)	12.6 (4.4–20.7)	0.12	0.63	0.063
**ENA -78**	35 (8.6–61.6)	107.7(42.2–173.1)	125 (39–211.4)	**0.015**	0.88	**0.020**
**Haptoglobin**	1664.6(905.1–2424.1)	845(295.2–1398.7)	935.2(181.91–688.4)	**0.029**	0.81	0.076
**IL-17A**	13.4 (9–17.9)	8.2 (4.7–11.7)	9 (3.6–14.3)	0.064	0.74	0.16
**IL-23**	0.81 (0.36–1.25)	0.38(0.036–0.72)	0.35 (-0.1–0.81)	0.083	0.83	0.17
**IP-10**	70 (24.3–115.4)	113.4(67.7–159.1)	137.6 (59.3–216)	0.11	0.49	**0.040**
**Lipocalin-2**	1415.5 (750.2–2080.8)	1393.2 (749.3–2037.1)	1097.6(425.58–1769.646)	0.62	0.053	**0.027**
**MIP-1 β**	16.2 (10.1–22.3)	17.6 (10–25.3)	23.7 (11.3–36)	0.38	0.098	**0.022**
**SDF-1a**	421.2 (324.4–518)	275.4 (151.7–399)	333.5 (164–503.1)	0.080	0.51	0.33
**Transthyretin**	1810.9(1577.4–2044.5)	1817.6(1611.4–2023.9)	1922.2 (1686.4–2158)	0.75	**0.047**	0.12
**VEGF**	355.5 (261.4–450)	392.2(304–480.4)	447 (359–535)	0.18	0.30	**0.034**

Data were analysed using mixed model repeated measures analysis of variance, with Fisher’s Least Significant Difference post hoc testing. The mean values shown (95% confidence intervals in brackets) are the least squared means. Significant P-values are in bold

## Discussion

In the present study, we evaluated the potential usefulness of 69 host markers as candidates for the salivary immunological diagnosis of TB disease and monitoring of the response to TB treatment. Although significant differences were observed in the concentrations of some of the markers including A2M, SAP, IL-17A, IL-1β, ECM1, IL-16, IL-23 and granzyme B, our study confirmed the notion that optimal diagnosis of TB disease may only be possible using biosignatures containing multiple analytes, as opposed to single markers. The most optimal biosignature identified in the present study was a five-marker signature comprising of IL-1β, IL-23, ECM-1, HCC1 and fibrinogen, which diagnosed TB disease with a sensitivity of 88.9% and specificity of 89.7%, regardless of HIV infection status. After excluding the HIV positive individuals, eight-marker biosignatures diagnosed TB disease with both sensitivity and specificity up to 100%, depending on the markers in the biosignature.

TB immunological tests may be more beneficial especially in resource-constrained settings, as they may be easily converted into point-of-care screening tests. Such tests would yield the highest impact if based on host markers that are detectable in easily available samples such as saliva, and are preferably based on the lateral flow technology. Out of the 69 host markers investigated in the current study, fibrinogen, antithrombin III, A2M, SAA, SAP, IL-1β, IL-23, IL-21, IL-13, IL-12(p40), HCC1, ENA-78, GDF-15, myoglobulin, TPA, granzyme A, granzyme B, PAI-1, α-1-antitrypsin, and complement C3, featured in diagnostic biosignatures for TB disease, thereby indicating that they may be candidate biomarkers for inclusion into future salivary diagnostic validation studies. Although most of these proteins are well-known and have been investigated widely in other diseases including TB [20–23], most of them have not previously been investigated in saliva samples, especially in the context of active TB disease.

In a previous small study comparing the expression of host markers in saliva to serum, vast differences were observed in the expression of host markers in saliva and serum, with some salivary host markers showing potential as diagnostic candidates [12]. In a relatively larger follow-up study [11], some of the potential host markers identified in [12] continued to show promise, with new host markers, and also markers showing potential as TB treatment response candidates identified [11]. IL-1β and IL-13 were amongst the markers that were more abundantly expressed in saliva, and IL-1β was included in top salivary biosignatures for the diagnosis of TB disease [12], as also observed in the current study. In addition to these two cytokines, other markers that showed potential in the present study as determined by p-values for differences between TB and ORD or inclusion into top biosignatures, and which were also identified in the two previous saliva based studies [11,12] as diagnostic candidates including fibrinogen, A2M, CRP, IP-10, MIP-1β, IL-17, VEGF and SAP, may be strong candidates for further investigation in future larger studies. Despite the agreement observed between the current and the two previous saliva based studies[11,12], we observed some differences between the current, and these previous studies. Although A2M and SAP were identified as important diagnostic candidates in this and the previous studies[11,12], we observed higher levels of A2M and SAP in saliva samples from individuals with ORD in the present study, in comparison to higher levels of these markers in TB patients in one of the relatively larger previous studies [11]. Although it may not be possible to completely explain the reasons for these differences, the use of limited numbers of study participants and the real potential for false discoveries exist, as patients used in these studies were not homogenous. The study by Phalane et al [12]was a small case-control study which was primarily aimed at investigating whether there were any differences in the expression of host markers between saliva and serum samples. The previous study by Jacobs et al [11] was a relatively larger study that was conducted on individuals that presented with signs and symptoms of TB, who were consecutively recruited, much like the study participants included in the current study. However only culture positive TB patients (n = 18), four of whom were HIV infected and randomly selected individuals with ORD were included in the current study, due to high costs associated with investigating the relatively large numbers of host markers in this study. Although the findings from all these studies require careful interpretation because of the heterogeneity in the studies, the potential host markers so far identified through these studies may be regarded as candidate biomarkers, requiring validation in larger studies. We also identified new salivary diagnostic candidate markers in the current study including IL-16, IL-23 and ECM-1.

IL-16 is a pro-inflammatory cytokine and a ligand for CD4^+^ T lymphocytes [24] and in the context of HIV, has been shown to inhibit the replication of the virus, with serum levels decreasing with HIV disease progression [25]. IL-23 is important in the Th17 pathway in *M*.*tb* infection, and is generally produced by alveolar macrophages and dendritic cells [25]. ECM-1 is a glycoprotein and plays a role in angiogenesis, and has been implicated in tumor progression [26,27]. IL-23 was amongst the markers included in the optimal five-marker signature identified in the present study and also showed promise individually as a diagnostic candidate for TB disease. Although not expressed at high levels, the concentrations of IL-23 and ECM1 were significantly higher in TB patients, with IL-16 levels higher in patients with ORD in the current study. These markers warrant inclusion into analyte panels in future validation studies.

We also investigated salivary levels of newly identified serum and plasma diagnostic host markers including NCAM, MIP-4 [28], transthyretin, Apo A-1 amongst others [17]. The levels of Apo A-1, NCAM and MIP-4 were not detectable in saliva samples in the current study, whereas transthyretin levels were not significantly different between TB patients and individuals with ORD, even though transthyretin showed potential as a marker of TB treatment response. Although salivary GDF-15 levels showed a trend towards higher levels in TB patients, the data obtained in the current study indicates that most of these markers may not be useful as diagnostic candidates in saliva, and require further investigation.

In addition to being potential salivary TB diagnostic candidates, Il-17A, IL-23 and ECM-1 also showed potential as markers for TB treatment response. The high concentrations observed for these markers in TB patients in comparison to ORD at baseline, reduced (although not always significantly) in the course of TB treatment. Our observations for CRP, IP-10 and VEGF as salivary candidate markers for monitoring of the response to treatment are in agreement with previous investigations [11,29,30]. As discussed previously [11], larger studies are required to validate the potential of these markers as biomarkers for TB treatment response. Such studies should include samples collected at earlier time points, for investigation of markers of early TB treatment response, and should also investigate whether these markers are informative in the prediction of month 2 culture outcome, favourable treatment outcome (clinical cure) and poor outcomes (relapse and treatment failure).

The main limitation of our study was the small sample size. As this was largely a discovery study, this might not be a major problem especially as we also confirmed findings from previous studies [11,12]. The few discrepancies between the markers identified in this and previous studies [11,12], strengthen the argument for the need for further validation of these candidate markers in larger studies before they may be considered for incorporated into point-of-care lateral flow test platforms. Although incorporation of host markers identified in the laboratory using platforms such as the Luminex technology into point-of-care tests is challenging, recent advances in technology have made such tasks possible. A prototype of such a point-of-care test was recently developed and successfully investigated in a recent Pan-African study [5], with further development of the platform ongoing (www.screen-tb.eu). The strength of such a test, based on validated biomarkers including the ones identified in the present study include non-invasive sampling, and such a test might be very useful in the diagnosis of paucibacillary TB. The effect of HIV infection and other comorbidities such as diabetes mellitus on salivary biomarkers requires investigated in future studies. Furthermore, such studies should also include individuals with confirmed other diseases that present with symptoms that are similar to TB including pneumonia.

## Conclusions

In conclusion, we have identified candidate salivary biosignatures with potential in the diagnosis of TB disease and monitoring of TB treatment response. Our findings require further validation in larger studies, before being considered as candidate markers for point-of-care diagnostic tests.
